# New Antimicrobial Peptide with Two CRAC Motifs: Activity against *Escherichia coli* and *Bacillus subtilis*

**DOI:** 10.3390/microorganisms10081538

**Published:** 2022-07-29

**Authors:** Olga Koksharova, Nina Safronova, Antonina Dunina-Barkovskaya

**Affiliations:** 1 Belozersky Institute of Physico-Chemical Biology, Lomonosov Moscow State University, Leninskie Gory, 1-40, 119991 Moscow, Russia; safronova.nina2007@mail.ru; 2 Institute of Molecular Genetics of National Research Center “Kurchatov Institute”, Kurchatov Square, 2, 123182 Moscow, Russia

**Keywords:** antibacterial activity, bacteria, *Bacillus subtilis*, cholesterol-recognition amino acid consensus motif (CRAC), *Escherichia coli*, peptides

## Abstract

Due to the emergence of multiple antibiotic resistance in many pathogens, the studies on new antimicrobial peptides (AMPs) have become a priority scientific direction in fundamental and applied biology. Diverse mechanisms underlie the antibacterial action of AMPs. Among them are the effects that AMPs cause on bacterial cell membranes. In this work, we studied the antibacterial activity of a peptide named P4 with the following sequence RTKLWEMLVELGNMDKAVKLWRKLKR that was constructed from two alpha-helical fragments of the influenza virus protein M1 and containing two cholesterol-recognizing amino-acid consensus (CRAC) motifs. Previously we have shown that 50 μM of peptide P4 is toxic to cultured mouse macrophages. In the present work, we have found that peptide P4 inhibits the growth of *E. coli* and *B. subtilis* strains at concentrations that are significantly lower than the cytotoxic concentration that was found for macrophages. The half-maximal inhibitory concentration (IC50) for *B. subtilis* and *E. coli* cells were 0.07 ± 0.01 μM and 1.9 ± 0.4 μM, respectively. Scramble peptide without CRAC motifs did not inhibit the growth of *E. coli* cells and was not cytotoxic for macrophages but had an inhibitory effect on the growth of *B. subtilis* cells (IC50 0.4 ± 0.2 μM). A possible involvement of CRAC motifs and membrane sterols in the mechanism of the antimicrobial action of the P4 peptide is discussed. We assume that in the case of the Gram-negative bacterium *E. coli*, the mechanism of the toxic action of peptide P4 is related to the interaction of CRAC motifs with sterols that are present in the bacterial membrane, whereas in the case of the Gram-positive bacterium *B. subtilis*, which lacks sterols, the toxic action of peptide P4 is based on membrane permeabilization through the interaction of the peptide cationic domain and anionic lipids of the bacterial membrane. Whatever the mechanism can be, we report antimicrobial activity of the peptide P4 against the representatives of Gram-positive (*B. subtilis*) and Gram-negative (*E. coli*) bacteria.

## 1. Introduction

Today, one of the biggest challenges facing modern medicine and public health is the emergence and rapid growth of antibiotic-resistant bacterial strains. The search for alternative drugs has shown that antimicrobial peptides (AMPs) can be considered as a promising alternative to antibiotics [1,2,3,4,5,6,7,8]. AMPs are known as amphipathic peptides that usually contain cationic and hydrophobic domains, which have antibacterial activity. AMPs are produced by many bacteria and are also produced in response to microbial invasion into various multicellular organisms (invertebrates and vertebrates, as well as plants) and, therefore, are one of the most important natural components of the humoral immune system [1,2,3,4,5,6,7,8]. In addition to naturally occurring AMPs, there are peptides with comparable biological activity that have been constructed with natural amino acid sequences or created de novo [9,10,11,12]. Currently, there are various databases of AMPs [13,14]; for example, the Antimicrobial Peptide Database (https://aps.unmc.edu/ accessed on 28 July 2022 ), which contains over 3300 AMPs from organisms of the six kingdoms of life [13], and the Database of Antimicrobial Activity and Structure of Peptides (DBAASP), which provides detailed information on the chemical structure and activity of more than 19,208 experimentally tested AMPs [14,15].

It is believed that the main mechanism of action of AMPs is related to the formation of pores in the membranes. Peptides create pores in the pathogen membrane, which leads to membrane depolarization and/or cell lysis [1,3,8,16,17,18,19]. Despite the large amount of data that have been obtained on cellular and model systems, the relationship between the structure of AMPs and their effect on cell membranes, as well as the basis for the selectivity of AMPs for specific target cells, remains a subject of debate. The ability of AMPs to destabilize the membrane depends on the interaction of peptides with the bacterial plasma membrane and on the composition of the membrane, which varies significantly in different bacterial species [20,21,22,23]. The action of antimicrobial peptides is pleiotropic and involves various mechanisms, which complicates the development of bacterial resistance to the peptide. Resistance to peptides would require major changes in the lipid organization of the bacterial membrane, which can occur gradually and can be prevented or corrected, unlike mutations underlying bacterial resistance to conventional antibiotics. This is an important advantage of AMPs over traditional antibiotics that explains the increase in the therapeutic use of antimicrobial peptides [24,25,26]. Another advantage of AMPs is the possibility of chemical modification of peptides, which improves their properties, for example, increases their proteolytic stability [27].

The membranes of many Gram-negative bacteria contain sterols, such as cholesterol or its derivatives and/or hopanoids that exhibit steroid-like properties and can form liquid-ordered lipid domains in bacterial membranes, just as cholesterol does in animal cells [20,21,22,23]. It was hypothesized that hopanoids are phylogenetic ancestors of sterols, which act as membrane enhancers in prokaryotic cells, in the same way as sterols do in eukaryotic membranes [22]. In Gram-negative bacteria, cholesterol plays an extremely important role in the organization and functions of membranes, due to its effect on the shape, mobility, and mechanical properties of membranes. Moreover, cholesterol affects the activity of many membrane proteins, such as receptors, enzymes, ion channels, and various transporters [28,29,30,31]. The membranes of Gram-positive bacteria contain no sterols. Despite the fact that membrane domains have also been observed in certain Gram-positive bacteria species, such as *Bacillus subtilis (B. subtilis)* and *Staphylococcus aureus (S. aureus),* their origin is still not clear and further work is necessary in order to clarify the physical and chemical bases underlying the formation of lipid raft domains in these bacteria [20].

It has been shown [32,33,34] that many cholesterol-dependent proteins have a motif that is called the “cholesterol-recognition amino acid consensus” (CRAC). The formula for this motif is V/L-X(5)-Y/W-X(5)-R/K, where X is any amino acid [32]. Subsequently, it was demonstrated by using various methods that peptides with CRAC motifs are able to interact with cholesterol and affect the cholesterol-dependent processes in cells [35,36]. Many AMPs contain CRAC motif(s) [37,38,39,40] and the activity of these AMPs depends on the intactness of these CRAC motifs, which may indicate the participation of sterols in the mechanism of AMP action. This aspect is not always taken into account in studies that are devoted to AMPs, although the presence of CRAC motifs in many AMPs is obvious (see, for example, [41,42,43,44]). 

Earlier, we have shown that a peptide RTKLWEMLVELGNMDKAVKLWRKLKR (named P4) that is constructed from two alpha-helical fragments of the influenza virus protein M1 and containing two CRAC motifs, modulates the cholesterol-dependent activity of mouse cultured macrophages IC-21 and exerts a cytotoxic effect at a concentration of 50 μM [45,46]. The aim of this work was to test the antibacterial activity of peptide P4 against representatives of Gram-negative bacteria and Gram-positive bacteria, *Escherichia coli (E. coli)* and *B. subtilis*, respectively, and to determine the role of CRAC motifs in the action of the studied peptide. 

## 2. Materials and Methods

### 2.1. Peptides and Peptide Stock Solutions

Peptides P4 and nScr were synthesized in Syneuro LLC (Syneuro Co. Ltd., Moscow, Russia). The primary structure of peptide P4 is Ac-RTKLWEMLVELGNMDKAVKLWRKLKR-NH_2_ and the sequence of nScr is Ac-WVGMALENRKLKKDRLKVLKMLRWT-NH_2_ (Supplementary, Table S1). Peptide P4 (26 a.a., mol. weight 3284 g/mol) contains two α-helix CRAC-containing peptides (CRAC motifs are underlined): LEVLMEWLKTR and NNMDKAVKLWRKLK (α-helix 3 of the M1 influenza virus protein, “peptide 1” from [47], and α-helix 6 of the M1 influenza virus protein, modified “peptide 2” from [47], with a replacement of tyrosine for tryptophan, see [45,46]). In peptide P4, the α-helical regions of the two constituent peptides are connected by a flexible loop that is formed by interhelical unstructured regions. The nScr peptide (“new scramble”) consists of the same amino acids as P4 peptide, but in random order, and contains no CRAC motifs. 

Before the start of experiment, the stock solutions of peptide P4 and peptide nScr were prepared. Each peptide was weighted and dissolved in dimethyl sulfoxide (DMSO, MP Biomedicals, France) and for each peptide a set of stock solutions with a concentration range of 0.2–5 mM was made (in its dissolved form, the peptide retains activity for at least 2 weeks, while being stored in the refrigerator (4 °C)). In the experimental setting, in order to obtain the final experimental concentration range of each peptide in the sample sets (0.01–10 μM of peptide for bacteria, 0.5–50 μM of peptide for macrophages) a certain amount from each peptide stock solution was added to each congruous sample in the sample set, which already contained bacteria culture or macrophages (see Section 2.3): the final concentration of DMSO in the incubation medium volume did not exceed 1%; bovine serum albumin (BSA, Sigma, Burlington, MA, USA) was added to all samples at a final concentration of 1 mg/mL. The control sample was a mixture of cells, incubation medium, 1% DMSO, and 1 mg/mL BSA, and did not contain the peptide. It should be noted that albumin itself at a concentration of 1 mg/mL stimulated the growth of *E. coli* and *B. subtilis* cells by up to 20–80% in comparison with conditions without BSA; we took this into account, but we did not analyze this effect of albumin and only compared cell growth parameters in the presence and absence of the peptide, with the other conditions being equal.

### 2.2. Bacterial Strains and Cultivation Conditions

The model bacterial strains of *E. coli* strain MC4100 and *B. subtilis* strain 168 were obtained from the collection of the Institute of Molecular Genetics RAS, Moscow, Russia. Bacterial strains were grown in Luria-Bertani liquid media broth (LB: 1% tryptone, 0.5% yeast extract, 0.5% NaCl) and on Petri dishes on agarized LA medium (LB with 1.5% agar) at 37 °C [44]. In our studies, medium M9 (0.6% Na_2_HPO_4_, 0.3% KH_2_PO_4_, 0.05% NaCl, 0.1% NH_4_Cl, after autoclaving 0.2% glucose, and 1 mL 0.1 M CaCl_2_ and 1 mL 1 M MgSO_4_ × 7 H_2_O per 1 L of medium were added) was used as an incubation medium [48]. Stationary-phase cultures of bacteria were obtained by growing the bacteria cells overnight (for approximately 10–14 h) in Luria-Bertani liquid media broth at 37 °C at a shaking rate of 150 rpm. The exponentially growing cells of the bacteria were obtained by diluting the overnight culture to a medium ratio of 1:20 and by growing them in LB medium for 1.5 h at 37 °C at a shaking rate of 150 rpm.

### 2.3. Treatment of Bacterial Cells with Peptides

The effect of the studied peptides on the bacterial viability was tested according to the method that was previously described in [49]. An overnight culture grown in Luria-Bertani broth was diluted 20-fold in fresh LB medium and cultured for 1.5 h at 37 °C at a shaking rate of 150 rpm. Then, the exponentially growing cells of each bacterial culture were washed from the LB medium 3 times by using the minimal medium M9. The concentration range was chosen based on the results that were obtained earlier on macrophages [45,46]. In order to prepare 1 mL of each bacterial experimental suspension that would contain the peptide (P4 or nScr) in the concentration range from 0.01 to 10 μM, an appropriate aliquot was taken from each prepared peptide stock solution (0.2–5 mM) and added to a corresponding volume of a mixture that contained M9 medium, 1 mg/mL BSA, and 100 μL of bacterial culture (cell density 10^7^ cells/mL). The control cell suspensions contained 1 mg/mL BSA and 1% DMSO and 100 μL of bacterial culture in the absence of peptide. All the prepared cell suspensions (experimental and control) were thoroughly mixed on a vortex (Vortex Grant Bio PV-1, Wiltshire, UK). The prepared suspensions were incubated for 1 h at 37 °C at a shaking rate of 150 rpm. The survival of bacteria after peptide treatment was determined by the CFU method, that is serial dilutions of aliquots of bacterial suspension in liquid M9 medium followed by plating on solidified agar medium LA. The experiments were performed in six technical replicates. The bacteria were grown on plates for 10–15 h in a thermostat at 37 °C. The number of colony-forming units (CFU, number of cells/mL) was counted for each experiment variant and compared with the control variant (in which bacteria were incubated in M9 medium in the absence of peptide and in the presence of 1 mg/mL BSA and 1% DMSO). The experiments were performed in three biological replicates. Statistical data processing (the calculation of the mean and standard deviation) was performed by using Microsoft Excel 2002, OriginPro 7.5 (OriginLab Corporation, Northampton, MA, USA), and GraphPad 9.3.1 software (GraphPad Software, LLC, San Diego, CA, USA). 

### 2.4. Evaluation of the Effects of the Peptides on Cultured Mouse Macrophages IC-21

The experiments were performed on mouse cultured peritoneal macrophages IC-21 (ATCC number TIB-186™) as described previously in [45,46,47]. The effect of peptides on cell phagocytic activity was determined by the number of fluorescently labeled 2 µm latex microspheres (Fluoresbrite Carboxy YG 2.0 Micron Microspheres, Polysciences, Inc., Warrington, PA, USA) that were associated with the cells. Cells in 6-well plates were preincubated at 37 °C in a CO_2_ atmosphere for 1 h in serum-free DMEM medium [50], then 1 mg/mL albumin, test peptide or DMSO at appropriate concentration (control), and fluorescently labeled particles (8 × 10^6^ particles per well) were added to the cells, and the cells were then incubated at 37 °C in a CO_2_ atmosphere for 1 h. In some experiments, to estimate the role of the membrane cholesterol depletion [51,52,53], 5 mM of methyl-β-cyclodextrin (mβCD, Sigma, Burlington, MA, USA) was added to the cells during the 1-h preincubation in serum-free DMEM. After subsequent incubation in the presence of test peptides, the cells were washed three times with phosphate buffered saline (PBS) to remove free and weakly bound particles and fixed with 2.5% glutaraldehyde solution (Ted Pella, Redding, CA, USA) in PBS. The fixed cells were examined using a Zeiss Axiovert 200 M fluorescence microscope (Carl Zeiss, Oberkochen, Germany) that was equipped with an ORCAII-ERG2 digital video camera (Hamamatsu, Shizuoka, Japan) and an appropriate software package (Axiovision 4.5, Carl Zeiss Imaging). In each well, 10–15 randomly selected fields of view were photographed in phase contrast mode, as well as in particle and glutaric aldehyde fluorescence mode (excitation/emission at 490/520 and 520/590 nm, respectively). The number of particles that were bound to the cells was determined using a specially developed software module of the ImageJ program [45,46,47,50]. The average number of particles per cell for a given well (“phagocytosis index”) was a parameter characterizing the cell activity. At least 200 cells in each well were used to estimate the phagocytosis index. The data are presented as the mean ± SE (standard error). The toxic effect was assessed by morphological criteria indicating cell damage (membrane fragmentation, nuclear boundary contrast, cell retraction, and rounded shape) and quantified as a percentage of destroyed cells in the field of view. Graphs were plotted using GraphPad Prism 9.3.1. (GraphPad Software, LLC, San Diego, CA, USA) and OriginPro 7.5 (OriginLab Corporation, Northampton, MA, USA).

### 2.5. Statistical Analysis

The mean and the standard deviation were calculated for each sample based on its replications. The data were subjected to the one-way analysis of variance (ANOVA) test. The differences between the samples were determined by the two-tailed *t*-test after Bonferroni error correction was performed. The difference between the compared values was considered statistically significant at *p* ≤ 0.05. 

Statistical data processing—calculation of the mean and standard deviation—was performed by using Microsoft Excel 2002 (Microsoft Corporation, Washington, DC, USA) and OriginPro 7.5 (OriginLab Corporation, Northampton, MA, USA).

## 3. Results

### 3.1. The Effect of Peptide P4 on E. coli 

**Figure 1:**
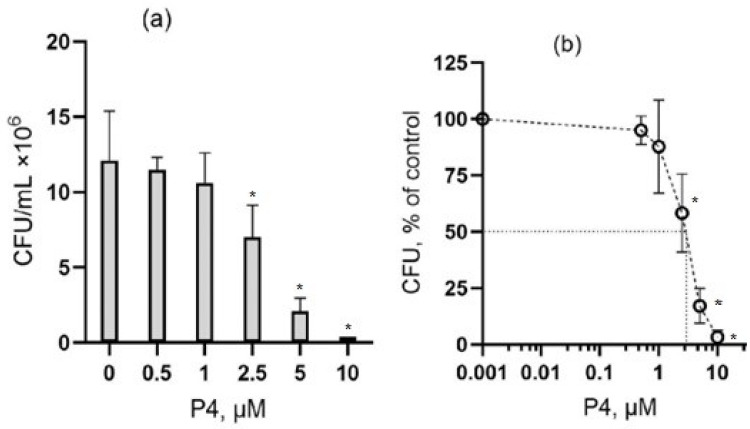
The dose-dependent effect of peptide P4 on the viability of Gram-negative bacteria *E. coli.* (**a**) The number of colonies (CFU, colony-forming units, number of cells/mL) that were found after incubation of exponentially growing bacterial cells in the presence of peptide P4 at a concentration range from 0 to 10 μM. All samples contained 1% DMSO and 1 mg/mL albumin (see section “Materials and Methods”). (**b**) The dose–response curve shows the effect of peptide P4 in the experiment illustrated in panel (**a**), the number of colonies that were found in the absence of the peptide was taken as 100%. * The difference from the control value is statistically significant at *p* ≤ 0.05.

In our experiments, it was found that peptide P4 has an antibacterial effect on Gram-negative bacteria *E. coli.* Hereafter, we define the lethal concentration as the lowest concentration that causes death to all cells, and the half-maximal inhibitory concentration IC50 as the concentration at which half of the exposed cells die. As it is shown in Figure 1, the survival of *E. coli* cells (*E. coli* strain MC4100) after 1-h incubation in the presence of peptide P4 is concentration-dependent. At the concentration level of 0.1 and 0.5 μM, there was no statistically significant difference (*p* > 0.05) in the number of colony-forming units (CFU) but at 5 μM, the number of CFU sharply decreased, and after 1-h incubation with 10 μM of peptide P4, no bacterial cell colonies were detected. So, the lethal concentration for peptide P4 was found in the range of 5–10 μM. In the experiment shown in Figure 1, the half-maximal inhibitory concentration (IC50) of peptide P4 was found to be 2.0 μM (Figure 1b) and the mean IC50 value that was found for three independent experiments was 1.9 ± 0.4 μM (mean ± standard deviation (SD), *n* = 3).

### 3.2. The Effect of Peptide P4 on B. subtilis

**Figure 2:**
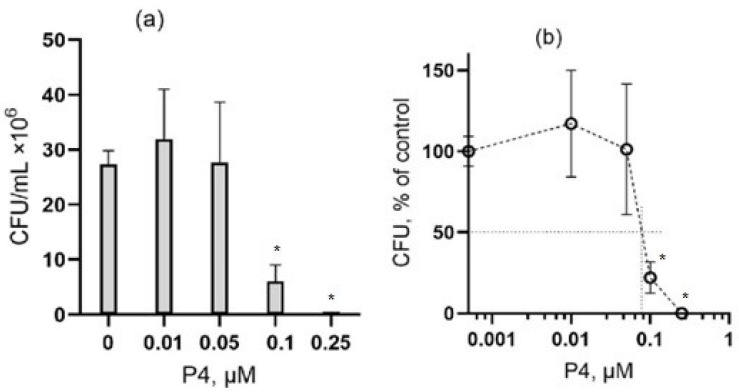
The dose-dependent effect of peptide P4 on the viability of Gram-positive bacteria *B. subtilis* (strain 168). (**a**) The number of colonies (CFU, colony-forming units, number of cells/mL) that were found after the incubation period of exponentially growing bacterial cells in the presence of peptide P4 at a concentration rate from 0 to 0.25 μM. All samples contained 1% DMSO and 1 mg/mL albumin (see “Materials and Methods”). (**b**) The dose-response curve shows the effect of peptide P4 in the experiment illustrated in panel (**a**); the number of colonies that were found in the absence of the peptide was taken as 100%. * The difference from the control value is statistically significant at *p* ≤ 0.05.

Peptide P4 also exhibited antibacterial activity against Gram-positive bacterium *B. subtilis*. The sensitivity of this bacterium to P4 treatment was significantly higher than that of *E. coli* after 1-h exposure. A noticeable decrease in CFU, which was found for *B. subtilis*, was observed already at a peptide concentration of 0.1 μM, and cell growth was completely suppressed at 0.25 μM (Figure 2). In this experiment the IC50 for peptide P4 was 0.07 µM (mean IC50 was 0.07 ± 0.01 μM, *n* = 3 independent experiments), which is more than an order of magnitude lower than the IC50 that was detected for *E. coli*.

### 3.3. The Effect of the Scramble Peptide (nScr) on Bacteria

**Figure 3:**
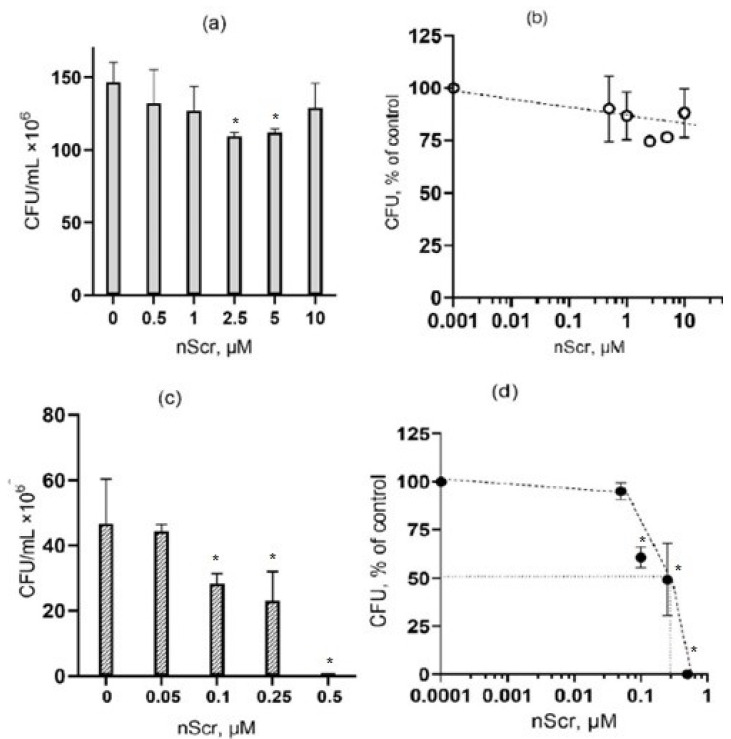
The effects of the “scramble” peptide nScr on the viability (colony-forming activity) of the exponentially growing *E. coli* (**a**,**b**) and *B. subtilis* (**c**,**d**) cells after 1-h exposure. Representative experiments are shown. Graphs in (**b**,**d**) are the peptide nScr dose–response curves that were plotted for the experiments that are illustrated in panels (**a**,**c**), respectively; the number of colonies in the absence of the peptide is taken as 100%. * The difference from the control value is statistically significant at *p* ≤ 0.05.

Peptide P4 contains two cholesterol-binding motifs, therefore, the antibacterial effect of P4 can be explained by its effect that leads to the change in membrane permeability that could be caused by the sequestration of cholesterol or other sterol-like lipids. Therefore, we tested the effect of the nScr peptide (“scramble”), which contains the same amino acids that make up peptide P4, but in a random order, so that CRAC motifs are absent. Our experiments have shown that the nScr peptide does not exhibit antibacterial activity against *E. coli* cells in the concentration range from 0.25 to 10 μM (Figure 3a,b). Interestingly, in the case of *B. subtilis* cells, the nScr peptide exhibited an antibacterial effect, although at a concentration that was higher than the concentration of peptide P4. In the experiment that is illustrated in Figure 3d, the half-maximal inhibitory concentration (IC50) was 0.4 μM for peptide nScr, which is six times higher than the IC50 of peptide P4 that was found for *B. subtilis* after 1-h incubation (see Figure 2b). In three independent experiments with *B. subtilis*, the mean IC50 value that was found for nScr was 0.4 ± 0.2 μM. The differences in the effects of peptides P4 and nScr on *E. coli* and *B. subtilis* are summarized in Table 1.

### 3.4. The Effects of Peptides P4 and nScr on Cultured Macrophages IC-21

**Figure 4:**
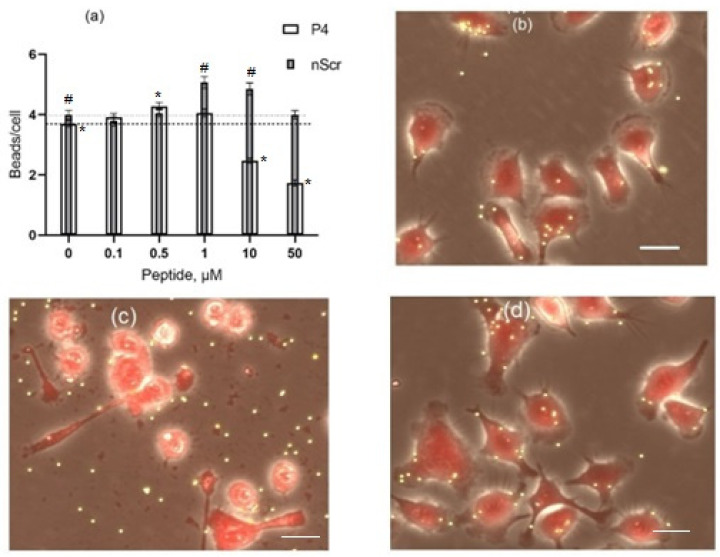
The effects of peptides P4 and nScr on cultured macrophages IC-21. (**a**) Dose-dependent effects of peptide P4 (white columns) and peptide nScr (gray columns) on cholesterol-dependent binding of 2-μm fluorescent beads by macrophages; error bars, SE; *, # the difference from the control value is statistically significant at *p* ≤ 0.05 (*, P4; #, nScr). The dashed line shows the control level for P4 and the dotted line, for nScr. (**b**–**d**) Micrographs of cells under control conditions (**b**), after incubation with 50 µM of peptide P4 (**c**), and with 50 µM of peptide nScr (**d**). The cytotoxic effect of 50 μM of peptide P4 can be seen in (**c**): all the cells are fragmented, the nuclei are contrasted, and most cells are retracted and have a rounded shape. Scale bar for (**b**–**d**) is 20 μm.

Previously, we have demonstrated that peptide P4 modulates the phagocytic activity of cultured macrophages IC-21 in a dose-dependent manner, and at a concentration of 50 μM peptide P4 was found to have a toxic effect. The phagocytic activity of macrophages was evaluated by the binding of 2-μm fluorescent microspheres [45,46]. In the current work we compared the effect that peptide P4 has on the activity and viability of macrophages with the effect that is caused by the «scramble» peptide nScr. Figure 4 illustrates the effects of these two peptides on macrophages IC-21 after 1-h of incubation of macrophage cells in the presence of peptides P4 or nScr in the concentration range from 0 to 50 μM. At the concentrations range from 0.1 to 1 μM, peptide P4 stimulated the binding of particles by the cells, and at 0.5 μM the number of particles per cell increased by 20% in comparison with the control (Figure 4a). At higher concentrations, the stimulating effect of the peptide was replaced by the suppression of cellular activity. At 50 μM, peptide P4 produced a robust cytotoxic effect; according to the morphological data, from 80 to 100% of macrophages cells were destroyed at this concentration (Figure 4c), and only 17 ± 9% (mean ± SD, *n* = 6), of the cells remained alive. This is consistent with our previous results [45,46].

Peptide nScr at a concentration range of 1–10 μM stimulated the activity of macrophages. However, unlike the effect found for peptide P4, the peptide nScr at a concentration of 50 μM did not suppress the macrophages cell activity (Figure 4a) and did not produce a cytotoxic effect (Figure 4d) after a 1-h treatment. This suggests that CRAC motifs play a key role in the mechanism of the cytotoxic action of peptide P4 and this finding is consistent with the results that were obtained earlier [46].

### 3.5. Cholesterol Extractant mβCD Lowers the Cytotoxic Concentration of Peptide P4 on Cultured Macrophages IC-21

**Figure 5:**
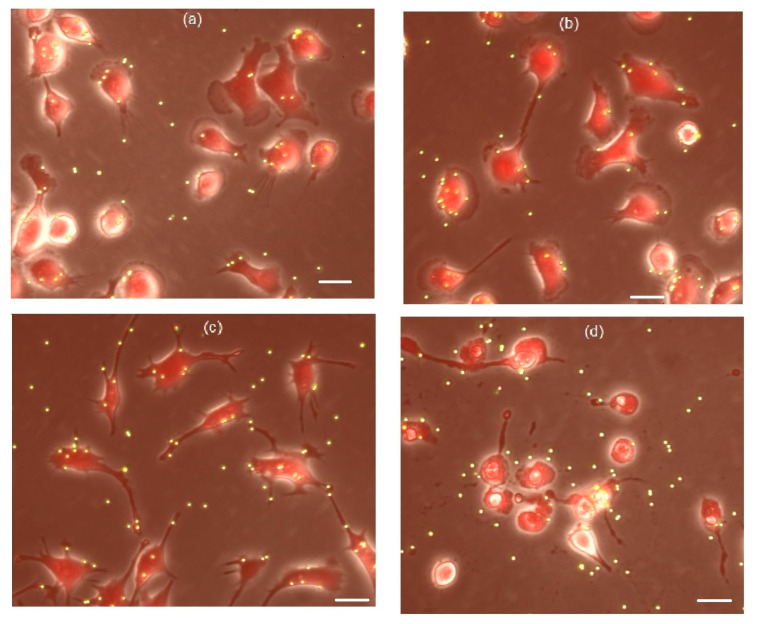
Cholesterol extracting agent mβCD reduces the cytotoxic concentration of peptide P4 for mouse cultured macrophages IC-21. Micrographs of macrophages IC-21 under control conditions (**a**), after 1-h incubation in the presence of 5 µM peptide P4 (**b**), 5 mM of mβCD (**c**), and in the presence of 5 µM peptide P4 after 1-h of pre-incubation with 5 mM of mβCD (**d**). After the cholesterol depletion with mβCD, the cells become much more sensitive to peptide P4, which exerts a toxic effect at 5 µM (**d**). 5 µM of peptide P4 (**b**) and 5 mM of mβCD (**c**), applied separately, are not toxic. The scale bar in (**a**–**d**) is 20 μm.

It was shown [45,46] that the sensitivity of cultured macrophages IC-21 towards the toxic action of peptide P4 significantly increases after the depletion of cell membrane cholesterol caused by a treatment with a cholesterol sequestering agent methyl-β-cyclodextrin (mβCD) [51,52,53]. Figure 5 illustrates an experiment in which cells were preincubated in the presence of 5 mM of mβCD before the treatment with 5 μM of peptide P4. No toxic effect or cell activity suppression were observed after the incubation of the cells with 5 mM of mβCD (Figure 5c; Supplementary Figure S1). Peptide P4 at a concentration of 5 μM was not toxic either (Figure 5b) and did not inhibit particle binding by macrophages (Supplementary Figure S2). However, the addition of 5 μM of peptide P4 to cells that have been pretreated with 5 mM mβCD lead to cell destruction, as can be seen in Figure 5d. This result indicates that a moderate withdrawal of cholesterol from cell membranes caused by mβCD, which does not affect the morphology of cells and their ability to bind particles, makes the cells much more sensitive to the toxic effect of the CRAC-containing peptide P4. This conclusion is consistent with our previous findings [45,46].

## 4. Discussion

In this work, we investigated the effect of a CRAC-containing peptide P4 on Gram- positive and Gram-negative bacteria. We found that peptide P4 demonstrates a strong antibacterial effect against the representatives of Gram-negative (*E. coli*) and Gram-positive (*B. subtilis*) bacteria. The sensitivity of *B. subtilis* cells to the P4 peptide was more than one order of magnitude higher than that of the *E. coli* cells. The half-maximal inhibitory concentrations (IC50) of peptide P4 were about 2 and 0.1 μM for *E. coli* and *B. subtilis*, respectively, after 1-h of exposure (Figure 1 and Figure 2). The cytotoxic effect of peptide P4 against mammalian cells (cultured mouse macrophages IC-21) was observed at 50 μM (Figure 4d), which is 5–10 times higher than the lethal P4 concentration for *E. coli* (5–10 μM) and more than two orders of magnitude higher than the lethal P4 concentration for *B. subtilis* (0.1–0.25 μM). Such a significant difference in the toxic doses of peptide P4 against bacteria and eukaryotic cells is certainly an important advantage of this AMP.

What is the possible role of CRAC motifs and cholesterol in the observed effects? We assume that CRAC motifs may play a role in the toxic effect of peptide P4 on macrophages and *E. coli* bacteria, as the membranes of these cells contain cholesterol. Moreover, the fact that the scramble peptide nScr lacking CRAC motifs was not toxic to these cells confirms this assumption.

We have previously shown that the cytotoxic effect of peptide P4 on macrophages is completely blocked when all the motif-forming amino acids are replaced by serine [46]. Moreover, the substitution of only aromatic acids in the CRAC motif (tryptophan) also inactivates the peptide [46]. The involvement of cholesterol in the mechanism of the toxic action on macrophages has been shown in experiments by using methyl-β-cyclodextrin (mβCD), an agent that extracts cholesterol from membranes [51,52,53]. Methyl-β-cyclodextrin is commonly used to modulate the cholesterol content in cell membranes and is recognized as a reliable tool for this purpose [51,52,53]. Experiments have shown that after treatment of mouse macrophages with mβCD, the cytotoxic concentration of peptide P4 decreased by an order of magnitude for macrophage cells (Figure 5, Supplementary, Figures S1 and S2; see also [45,46]). The fact that *E. coli* cells with low cholesterol content are more sensitive to peptide P4 than macrophages is consistent with this correlation (the lower cholesterol content, the higher the sensitivity of the cells to a CRAC-containing peptide).

This dependence of the toxic effect of a CRAC-containing peptide from the cholesterol membrane content can be supported by various mechanisms. For example, a CRAC-containing peptide can compete with cholesterol-dependent membrane proteins for binding to cholesterol and, by sequestering cholesterol, cause malfunctioning of these proteins, which can lead to cell death [53,54,55,56,57,58]. Consequently, the higher the cholesterol content is in the membrane, the higher the toxic concentration of the CRAC-containing peptide ought to be for the cells. In this case, cholesterol performs a membrane-protective function [29,30,31,53,54].

Another possible mechanism of the toxic effect of P4 depends on the ability of the peptide monomers to oligomerize in the membrane to form highly permeable pores without cholesterol involvement, which is characteristic of amphipathic peptides [30,31,44]; such pores can be detrimental to the cell. In this case, binding of membrane cholesterol with the CRAC-containing peptide monomers can prevent peptide oligomerization and pore formation and increase the toxic concentration of the peptide; in this situation cholesterol also acts as a membrane protector [54,55,59,60].

What could be the mechanism of the toxic effect of peptide P4 on *B. subtilis* that has no cholesterol and the toxic effect of peptide P4 cannot be explained by the interaction of P4 with cholesterol? Moreover, the “scramble” peptide nScr, which contains the same amino acids as peptide P4, but in a random order (i.e., without CRAC motifs), also proved to be toxic to *B. subtilis*.

We suppose that in this case, the essential factor is not the CRAC motif, but the cationic amino acids that are present in the amphipathic peptides P4 and nScr. These peptides, besides the motif-forming aliphatic and aromatic amino acids, contain eight cationic amino acids. These amino acids interact with anionic phospholipids, such as phosphatidylglycerol, phosphatidylserine, and cardiolipin, which are present in Gram-positive bacteria. This assumption is consistent with the results of Omardien et al. [61], who have demonstrated the toxic effects on *B. subtilis* cells of antimicrobial cationic peptides TC19 (LRCMCIKWWSGKHPK) and TC84 (LRAMCIKWWSGKHPK) containing CRAC motifs (underlined) and the bactericidal peptide BP2 (GKWKLFKKAFKKKFLKILAC) without a CRAC motif but containing motif-forming amino acids. The authors have shown that these peptides reduce the membrane potential and increase the permeability of the *B. subtilis* membrane and that this destabilizing effect is associated with a peptide-induced increase in the total area of fluid-disordered domains in the bacterial membrane. These domains can be formed as a result of the interaction of cationic peptides with anionic phospholipids. The question of whether the inhibitory effects of P4 and nScr on *B. subtilis* that were observed in our experiments are related to these interactions between peptides and anionic lipids requires further investigation. It is important to note that our work revealed two new peptides, P4 and nScr, with antibacterial activity against the Gram-positive bacterium *B. subtilis*, and there are good reasons to test the activity of these peptides against other Gram-positive bacteria in future experiments.

An analysis of published data shows that many antimicrobial peptides have CRAC motifs [41,42,43,44], although this is not always noted by the authors of the publications. For example, such motifs are present in protegrins and their derivatives, which represent a new class of peptide antibiotics based on mammalian antimicrobial peptides [41]. Protegrins contain 16–18 amino acids and well-defined CRAC motifs (e.g., protegrin 1, RGGRLCYCRRFCRVCVGR, and protegrin 4, RGGRLCYCRGWICFCVGR; CRAC motifs are underlined). Protegrins exhibit antimicrobial activity against Gram-positive and Gram-negative bacteria [41]. The presence of CRAC motifs is also evident in AMPs from frog skin, magainins (magainin 1, GIGKFLHSAGKFGKAFVGEIMKS; magainin 2: GIGKFLHSAKKFGKAFVGEIMNS) [42] and in some temporins [43,44]. For example, among the temporins that have been studied, temporin L containing the CRAC motif (FVQWFSKFLGRIL) has the greatest antibacterial activity against bacterial and fungal strains [44]. The lethal concentration of temporin L was 0.3 µM for *Bacillus megaterium* Bm11 and 1.5 µM for *E. coli* D21, which is an order of magnitude lower than the lethal concentration of temporin B (LLPIVGNLLKS) that does not contain the CRAC motif. In addition, temporin L caused erythrocyte hemolysis at a concentration of 50 μM and was cytotoxic to three human tumor cell lines. Based on their experiments with liposomes, the authors suggested that the disruption of bilayer organization was due to the formation of pores in the membrane [43,44].

It is noteworthy that the inhibitory concentrations of peptide P4 that were obtained in our work for Gram-negative and Gram-positive bacteria and mammalian cells are very close to the corresponding inhibitory concentrations of temporin L. In addition, Rinaldi et al. [44] noted the same pattern as we have observed in our current work regarding the cell sensitivity towards AMPs: cholesterol-free Gram-positive bacteria were more sensitive, while mammalian cells with a high cholesterol content in the plasma membranes were the least sensitive [28,29,30,31,61]. This may indicate the presence of a sterol-dependent component in the mechanism of bactericidal action of CRAC-containing AMPs: when the cholesterol content in the membrane is low, the AMP destabilizing effect develops at lower peptide concentrations. The membrane-protective properties of cholesterol against many toxic peptides have been noted in many works (e.g., [54,55,59,60]).

It should be added that the bactericidal effect of peptides can be exerted not only through the destabilization of the membranes or interference with the functioning of membrane proteins [56,57,58,61,62], but also through the interaction of AMP with other cellular targets. For example, it has been shown [63] that the antimicrobial effect of some proline-rich AMPs, in addition to the effect on membranes, also includes the blockade of protein synthesis due to the binding of these peptides to ribosomes, as it occurs in the case of macrolide antibiotics [64]. These mechanisms deserve a detailed study.

Thus, the results that were obtained in our work allow us to consider peptides carrying CRAC motifs as promising antimicrobial agents. A significant difference in the toxic doses of peptide P4, which are required against bacteria and eukaryotic cells, provides a good therapeutic window. Moreover, we found that at a dose that is toxic to bacteria, peptide P4 moderately stimulates the activity of macrophages; this means that peptide P4 can combine an antimicrobial effect and an immunostimulating effect. Further studies will be necessary to determine the working concentrations of this peptide in vivo. In addition, chemical modifications may be required to increase the proteolytic stability of the peptide (see [27] and references therein). Subsequent studies employing mutagenesis, transcriptomics, and proteomics methods can give us more answers and a better understanding of the molecular mechanisms underlying the antibacterial action of CRAC-containing peptides.

## 5. Conclusions

In this study, we demonstrated for the first time that the previously constructed peptide Ac-RTKLWEMLVELGNMDKAVKLWRKLKR-NH_2_ (named P4) with two cholesterol-recognition (CRAC) motifs exhibits antibacterial activity against representatives of Gram-positive (*B. subtilis*) and Gram-negative (*E. coli*) bacteria in the submicromolar and micromolar concentration range, respectively. The sensitivity of Gram-positive bacteria was 20 times higher than that of Gram-negative bacteria, and in general the sensitivity of these bacteria was more than an order of magnitude higher than that of cultured mammalian cells—mouse macrophages IC-21. Cholesterol depletion by using mβCD reduces the toxic dose of peptide P4 for macrophages, which indicates a cholesterol-dependent mechanism of the cytotoxic effect of CRAC-containing peptide P4. The scramble peptide nScr, devoid of CRAC-motifs, was not toxic to macrophages and *E. coli*, but was toxic to *B. subtilis*, lacking cholesterol, which suggests a cholesterol-independent mechanism of antibacterial effect of peptide P4 in the case of Gram-positive bacteria. The results obtained and the analysis of the literature indicate that the development of CRAC-containing AMPs is promising and potentially productive for the design of new antibacterial agents. Upcoming studies can help to clarify the detail mechanisms of antibacterial action of the new peptides P4 and nScr.

## Figures and Tables

**Table 1:** The antibacterial effects of peptides P4 and nScr on the cell growth of the model strains of *E. coli* and *B. subtilis*.

Strain	P4, IC50 ^1^ (μM)	nScr, IC50 ^1^ (μM)
*B. subtilis* strain168 (Gram-positive)	0.07 ± 0.01	0.4 ± 0.2
*E. coli* strain MC4100 (Gram-negative)	1.9 ± 0.4	no inhibition

^1^ Half maximal inhibitory concentration (IC50) is represented as mean ± standard deviation (*n* = 3).

## Data Availability

Not applicable.

## Supplementary Materials

The following supporting information can be downloaded at: https://www.mdpi.com/article/10.3390/microorganisms10081538/s1, Figure S1: Cholesterol depletion by mβCD dose-dependently modulates the ability of macrophages to bind 2-micron fluorescent particles; Figure S2: Cholesterol depletion by mβCD lowers the toxic concentration of P4 in cultured mouse macrophages IC-21; Table S1: Amino acid sequences and molecular weight of peptides P4 and nScr.
