# Supplementary material for: New Antimicrobial Peptide with Two CRAC Motifs: Activity against Escherichia coli and Bacillus subtilis
Source: Microorganisms. 2022 Jul 29;10(8):1538. doi: 10.3390/microorganisms10081538 (PMC9412426; doi:10.3390/microorganisms10081538)
Supplement: Supplementary file 1 [file microorganisms-10-01538-s001.zip › microorganisms-1822577-supplementary.pdf]

Supplementary Materials

Figure S1. Cholesterol depletion by methyl-beta-cyclodextrin (mβCD) dose-dependently modulates the ability of macrophages to bind 2-micron fluorescent particles. The cells were incubated for 1 h in the presence of different concentrations of mβCD and the mean number of cell-associated particles was calculated (see Methods; described in detail in [41, 42]). At a concentration of 20 mM mβCD strongly inhibits the cell activity but does not produce a toxic effect. (a, b) Dose-dependence of the effect of mβCD on particle binding by macrophages; panel (b) represents the experiment shown in (a) as a dose–response curve. (c) Micrographs of the cells have been taken after 1-h exposure to 0-, 5-, and 20-mM mβCD. At 20 mM the cells shrink and acquire a rounded shape; the processes of the cells become thinner and irregular.

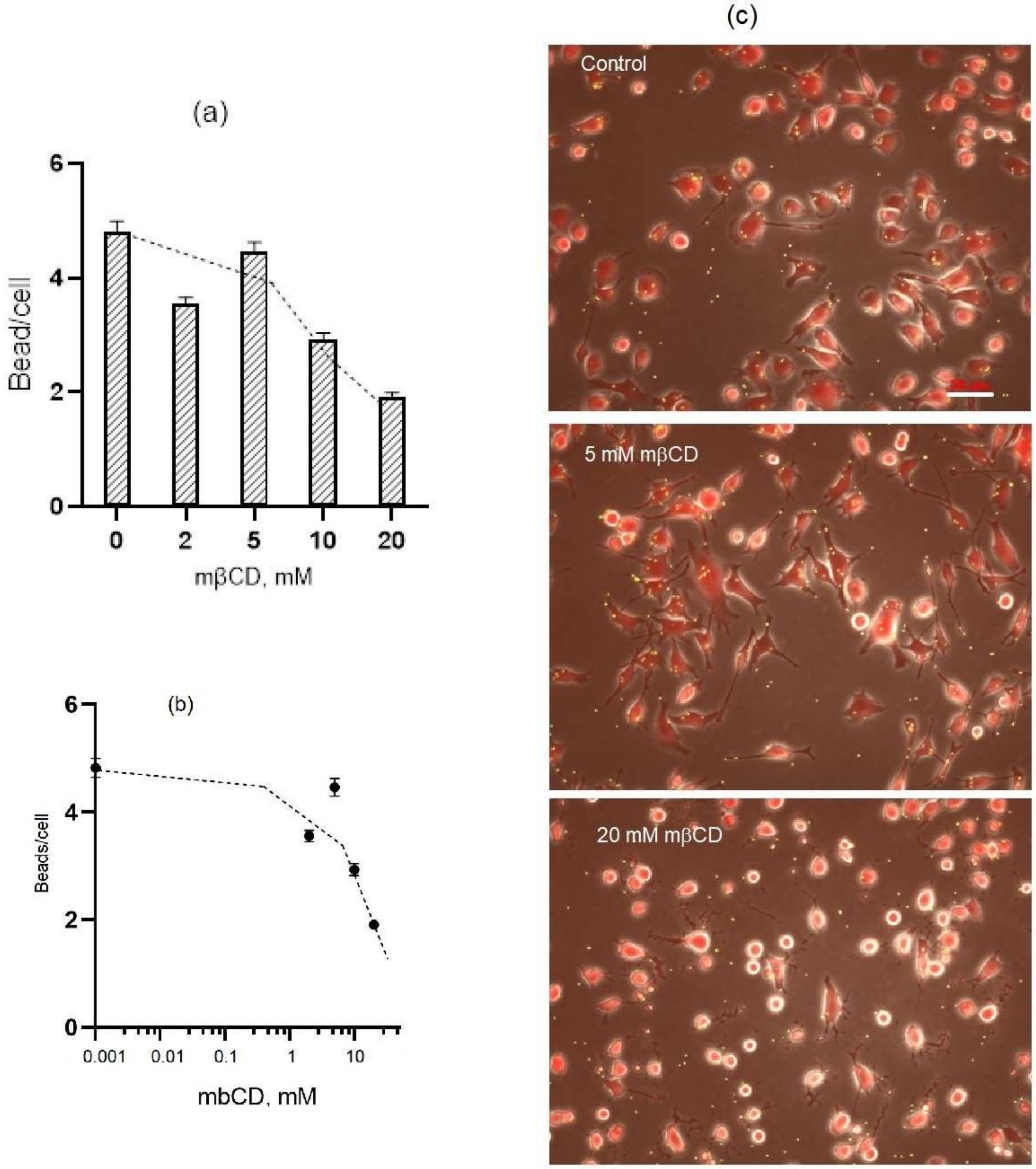

Figure S2. Cholesterol depletion by mβCD lowers the toxic concentration of P4 in cultured mouse macrophages IC-21. (a) Application of 5 mM mβCD or 5 µM P4 separately stimulates the activity of the cells; combined application of 5 mM mβCD and 5 µM P4 inhibits the cell activity. (b–e) Micrographs illustrating the effects of 5 mM mβCD and 5 µM P4: (b) control; separate application of 5 mM mβCD (c) or 5 µM P4 (d) does not produce a toxic effect, but combined application of 5 mM mβCD and 5 µM P4 (e) produces a cytotoxic effect. Scale bar, 50 µm.

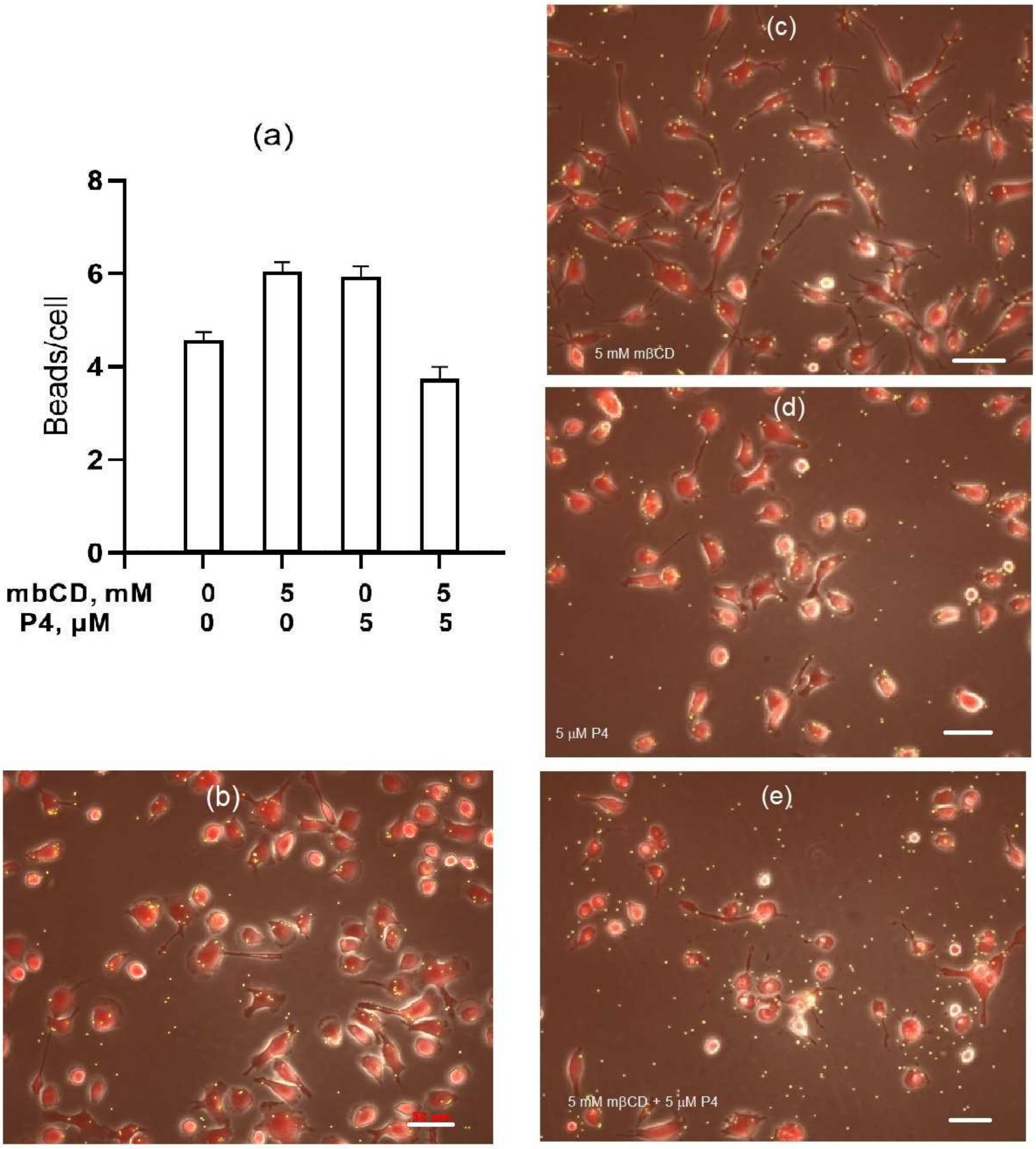

Table S1. Amino acid sequences and molecular weight of peptides P4 and nScr

Peptide	Sequence	Molecular mass g/Mol
P4	RTKLWEMLVELGNMDKAVKLWRKLKR	3284
nSCR	WVGMALENRKLKKDRLKVLKMLRWT	3155

Calculations were performed by using tools of Peptide 2.0 Inc
https://www.peptide2.com/peptide_molecular_weight_calculator.php

1 5 10 15 20 25
R T K L W E M L V E L G N M D K A V K L W R K L K R --- P4
W V G M A L E N R K L K K D R L K V L K M L R W T *E* ---- nScr
